# Bladder cancer immune microenvironment: immune-cell distribution, disease associations, treatment remodeling, and baseline immune features associated with treatment response and prognosis

**DOI:** 10.3389/fonc.2026.1985263

**Published:** 2026-10-27

**Authors:** Shaobo Qin, Xu Yu, Shishuai Zuo, Xiangfei He, Jie Liu

**Affiliations:** 1 School of Clinical Medicine, Shandong Second Medical University, Weifang, China; 2 Linyi People’s Hospital, Shandong Second Medical University, Linyi, China

**Keywords:** baseline biomarkers, BCG, bladder cancer, immune-cell localization, predictive-prognostic features, treatment-associated remodeling, tumor immune microenvironment (TIME)

## Abstract

Bladder cancer is characterized by substantial heterogeneity in the tumor immune microenvironment (TIME), which influences recurrence, progression, treatment resistance, and responses to Bacillus Calmette–Guérin (BCG), neoadjuvant chemotherapy, immune checkpoint inhibitors, and radiotherapy. This narrative review critically synthesizes evidence on immune-cell distribution, disease-state associations, treatment-associated remodeling, and baseline immune features linked to treatment response and prognosis. Current evidence indicates that the clinical meaning of immune infiltration depends not only on cell abundance but also on localization across histological compartments, functional state, tumor compartment, and therapeutic context. Cytotoxic lymphocyte infiltration may indicate antitumor immune activity when tumor-accessible and functionally active, whereas regulatory T cells, myeloid-derived suppressor cells, and M2-like tumor-associated macrophages often reflect suppressive or treatment-resistant immune states. Treatments can reshape the bladder cancer TIME, but the strength of evidence varies across modalities: BCG has relatively stronger human support for treatment-associated immune changes, whereas evidence for chemotherapy-, immunotherapy-, and radiotherapy-associated remodeling remains more heterogeneous. Baseline or pretreatment immune architecture may provide a more informative basis for biomarker development than single immune-cell markers. Future longitudinal studies with matched pre- and post-treatment specimens are needed to determine whether treatment-induced TIME changes provide additional treatment-response-associated or prognostic information beyond baseline immune features, whereas establishment of predictive utility will require comparative treatment designs with formal biomarker-by-treatment interaction testing.

## 1 Introduction

Bladder cancer is a common urinary tract malignancy characterized by high recurrence and progression rates. Approximately 75% of newly diagnosed bladder cancers are non-muscle-invasive bladder cancer (NMIBC), which is typically managed with transurethral resection of the bladder tumor followed by risk-adapted intravesical therapy such as Bacillus Calmette–Guérin (BCG). For muscle-invasive bladder cancer (MIBC), treatment usually involves radical cystectomy-based multimodal management; cisplatin-based neoadjuvant chemotherapy (NAC) is used in eligible patients, while immune checkpoint inhibitors (ICIs) have become important in selected perioperative or advanced/metastatic settings (1). However, treatment resistance remains a major bottleneck limiting survival benefits for bladder cancer patients. For example, a substantial proportion of high-risk NMIBC patients experience recurrence or progression after BCG therapy (2), and among MIBC patients receiving cisplatin-based NAC, only about 30% to 40% achieve pathological complete response (3).

This clinical resistance dilemma stems not only from intrinsic mutations in tumor cells but is also profoundly influenced by the TIME (1, 4). The tumor immune microenvironment (TIME) is a critical component of the tumor microenvironment(TME). The immune microenvironment of bladder cancer specifically refers to the dynamic, interactive network composed of diverse immune cell populations—which infiltrate the tumor and its surrounding areas and possess antitumor or immunosuppressive functions—and the factors they secrete (4). Bladder cancer exhibits strong immunogenicity, often accompanied by the infiltration of effector cells and the potential to respond to immunotherapeutic interventions (5). However, during actual tumor progression, highly concentrated populations of immunosuppressive cells within the TIME may profoundly alter the course of tumor development (4). Specifically, tumor-associated macrophages (TAMs) with phenotypes commonly described as M2-like contribute to immune evasion, angiogenesis, matrix remodeling, and malignant invasion; simultaneously, regulatory T cells (Tregs) and myeloid-derived suppressor cells (MDSCs) can suppress effector CD8^+^ T-cell activation and limit effective antitumor immunity, collectively contributing to a suppressive immune barrier (4). Furthermore, recent single-cell RNA sequencing (scRNA-seq) studies have revealed marked cellular heterogeneity within the bladder cancer microenvironment, highlighting the diversity of immune-cell transcriptional states (6). In parallel, histological and immunohistochemical studies indicate that the localization of immune cells across tumor nests, tumor stroma, invasive fronts, and other anatomical compartments may influence local immune activity and tumor behavior.

A thorough analysis of both baseline TIME architecture and treatment-associated immune changes is of great value for understanding clinical treatment resistance in bladder cancer (1). Several recent and foundational reviews have described immune components, immune-cell crosstalk, and immunotherapeutic strategies in bladder cancer (1, 4, 5). However, two related questions should be distinguished. One concerns how treatments such as BCG, NAC, ICIs, and radiotherapy are associated with changes in the TIME. The other concerns how baseline or pre-treatment TIME characteristics are associated with therapeutic response and prognosis. Separating these questions is important because treatment-associated immune changes are not automatically equivalent to validated predictive biomarkers.

Accordingly, the distinct contribution of this critical narrative review is to connect four layers of evidence: the histological compartmentalization of immune infiltration, the disease-state context of NMIBC and MIBC, treatment-associated remodeling of the TIME after major therapeutic modalities, and baseline immune features associated with treatment response and prognosis. In this framework, treatment-associated remodeling is discussed primarily as a mechanistic and descriptive theme, with recognition that the strength of evidence differs across treatment modalities. Prediction of treatment response and prognosis is discussed mainly in relation to baseline or pre-treatment TIME characteristics unless a cited study explicitly evaluates post-treatment immune changes and clinical outcomes.

Throughout this review, prognostic, treatment-response-associated, and predictive biomarkers are treated as distinct concepts. Prognostic biomarkers are associated with clinical outcomes irrespective of treatment, whereas treatment-response-associated biomarkers are associated with response or outcome within a treated cohort but do not by themselves establish treatment-specific benefit. Predictive biomarkers, in the strict sense, require comparative evidence demonstrating biomarker-by-treatment interaction. Accordingly, findings from single-arm or treatment-specific cohorts are described as treatment-response-associated unless treatment-effect modification has been demonstrated.

We also distinguish histological compartment-based localization from high-dimensional spatial profiling. The former refers to pathological or immunohistochemical assessment of immune cells across predefined anatomical compartments, whereas the latter uses multiplex or spatially resolved approaches that preserve tissue coordinates while characterizing multiple cellular or molecular features. Terminology is used according to the methodology of each study. Relevant literature was identified through PubMed, Web of Science, and Google Scholar, with primary emphasis on studies published between 2015 and 2025; earlier foundational studies were selectively included when directly relevant. Search terms covered bladder cancer/urothelial carcinoma, the tumor immune microenvironment, immune-cell populations, spatial localization, BCG, NAC, ICIs, radiotherapy, treatment response, and prognosis. Priority was given to original human and translational studies directly addressing the major themes of this review, while preclinical and conference-only reports were included when relevant and interpreted according to their evidentiary strength. Because this was a narrative review, no formal PRISMA-based screening or meta-analysis was performed.

## 2 Discussion

The following discussion is structured around four connected questions: first, how immune-cell populations are localized across histological compartments in bladder cancer tissue; second, how these immune patterns relate to tumor stage, grade, and invasiveness; third, how standard and emerging treatments remodel the TIME; and fourth, which baseline or pre-treatment immune features are associated with treatment response and prognosis. These latter two questions are intentionally separated: Section 2.3 summarizes treatment-associated TIME changes, whereas Section 2.4 focuses primarily on baseline or pre-treatment biomarkers and their clinical associations.

A recurring theme across the literature is that apparently similar levels of immune infiltration can have different clinical meanings. For example, CD8^+^ T-cell infiltration may be compatible with effective immune surveillance when tumor-accessible and accompanied by proliferation- or activation-associated features, but CD8^+^ cell density or individual marker expression alone does not establish cytotoxic activity. Likewise, PD-1/PD-L1 expression may be associated with exhaustion-related or adaptive immune-resistance phenotypes, but does not by itself demonstrate functional exhaustion. Therefore, baseline immune architecture, treatment exposure, and clinical endpoint should be interpreted separately rather than collapsed into a single biomarker concept. Similarly, cell identity, marker expression, inferred functional phenotype, and demonstrated biological activity should be regarded as related but non-equivalent levels of immune characterization.

### 2.1 Immune-cell localization across histological compartments in the bladder cancer TIME

The TIME in bladder cancer is not merely a phenomenon of single-cell infiltration, but rather a dynamic system composed of multiple immune cell types. These immune cells primarily include CD8^+^ T cells, CD4^+^ T cells, natural killer (NK) cells, Tregs, B cells, MDSCs, M2-like TAMs, and dendritic cells (DCs). Some of these immune cells exert effects such as antigen recognition, cytotoxic killing, and amplification of antitumor responses, while others exert immunosuppressive effects and promote tumor immune evasion. The compartmentalized organization of major immune-cell populations within the bladder cancer TIME is schematically illustrated in **Figure 1**. CD8^+^ T cells are one of the most critical cytotoxic effector cell populations in the bladder cancer TIME. They mediate tumor-cell elimination through cytotoxic effector mechanisms and cytokine-mediated immune activation (4, 5). However, the prognostic and predictive value of CD8^+^ T cells depends not only on their density but also on their localization and functional state. Studies of muscle-invasive and compartment-specific tumor-infiltrating lymphocytes have observed CD8^+^ T cells in both the tumor stroma and tumor nests, with non-uniform distribution across tumor compartments in some cohorts (7, 8). These differences suggest that CD8^+^ infiltration should not be interpreted as a binary immune-hot or immune-cold variable. A tumor may contain abundant CD8^+^ T cells yet remain clinically resistant when these cells are excluded from tumor nests or accompanied by exhaustion-associated marker patterns. However, localization or expression of individual checkpoint markers alone does not establish impaired cytotoxic function or functional exhaustion.

**Figure 1:**
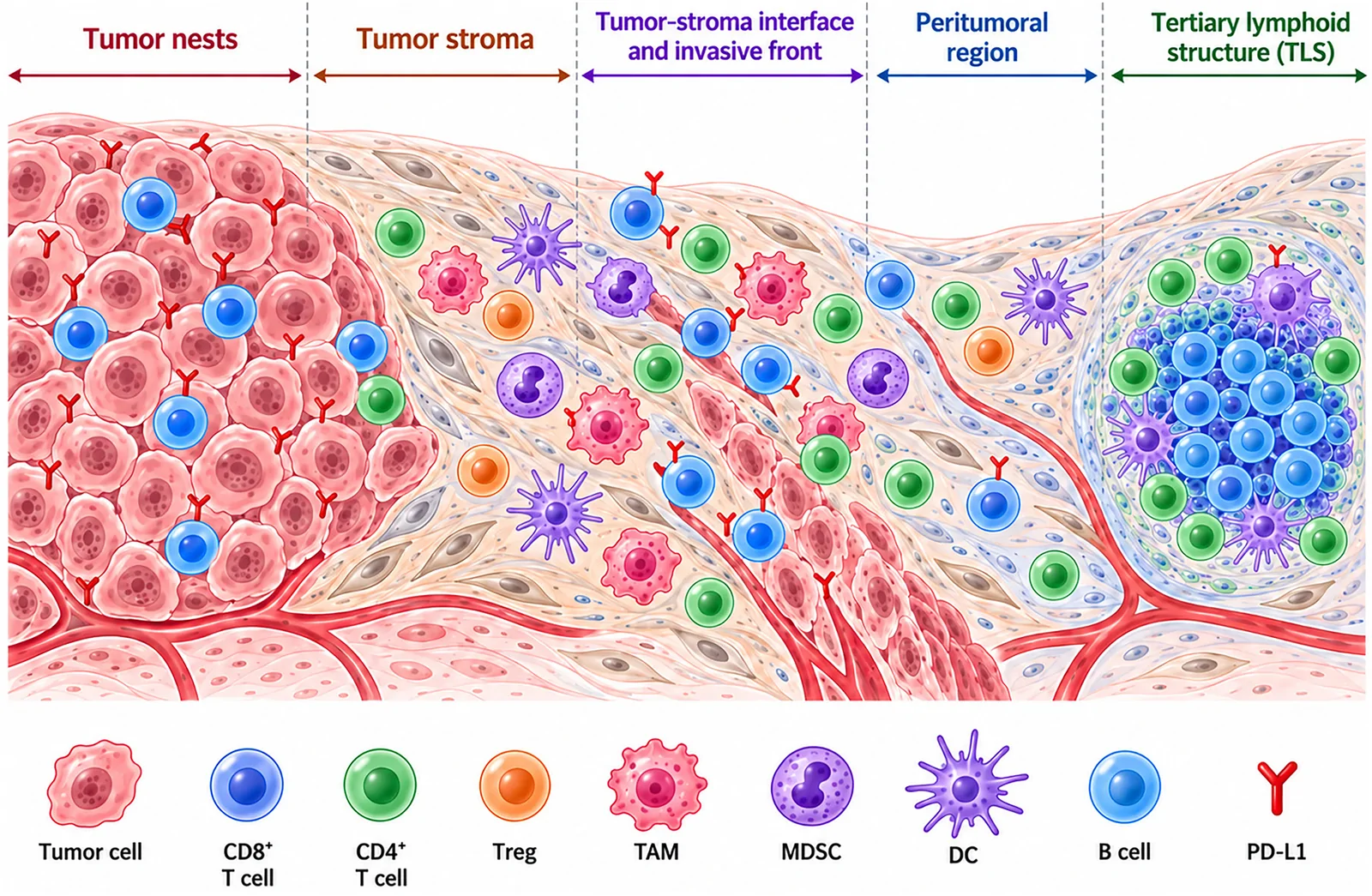
Bladder cancer tumor immune microenvironment as a spatial ecosystem. Schematic representation of immune-cell organization across representative histological compartments, including tumor nests, tumor stroma, the tumor–stroma interface, the invasive front, peritumoral regions, and tertiary lymphoid structures (TLS). Major immune-cell populations are shown with non-uniform distributions across these compartments. Cell positions are schematic and do not represent fixed or universal distributions.

CD4^+^ T-cell subsets further illustrate why functional state is as important as cell number. Although they are often described as co-regulators of CD8^+^ T-cell activation, CD4^+^ T cells include Th1, Th2, memory, cytotoxic, regulatory, and CXCL13-expressing populations with different effects on antitumor immunity (4, 9). Oh et al. found that CD4^+^ T cells, including CXCL13-expressing and cytotoxic CD4^+^ subsets, can be enriched within tumor tissue, whereas memory and activated CD4^+^ T cells may be more common in peritumoral regions (9). Pichler et al. reported a Th2-predominant intratumoral phenotype with a high GATA3/T-bet ratio, while also noting compartment-specific enrichment of CD4^+^ cells in the mucosal lamina propria and at the invasive front (10). In contrast to helper or cytotoxic CD4^+^ programs, Tregs form a suppressive regulatory axis through IL-10, TGF-β, CTLA-4, and PD-L1-related mechanisms (4). Their preferential localization in the tumor stroma or peritumoral region in NMIBC and other bladder cancer specimens suggests that Tregs may help create a physical and functional barrier between effector lymphocytes and tumor cells (11).

NK cells represent an innate effector axis in bladder cancer, but the evidence is heterogeneous, likely reflecting differences in tumor stage, histology, detection platform, and the specific NK markers used. Mukherjee et al. found intratumoral NK cells to be present in some bladder cancer tissues, with CD56bright NK cells associated with improved survival; later work further linked CD56 expression to NK-cell-mediated antitumor activity (12, 13). Single-cell RNA sequencing further suggests that NK-cell infiltration may be higher in MIBC than in NMIBC or adjacent normal tissue (6). Taken together, the NK-cell compartment should be treated as a context-dependent effector axis rather than a uniformly present feature of bladder cancer TIME.

Beyond these major lymphoid populations, several less abundant or less extensively characterized lymphoid subsets have also been identified in bladder cancer. Non-NK innate lymphoid cells (ILCs), particularly ILC1-like populations, have been detected within bladder tumors and can exhibit mixed inflammatory and Th17-like features, indicating functional plasticity within the local TIME (14). γδ T cells, including Vδ2 T cells, are present in bladder cancer tissue and peripheral blood and can recognize bladder tumor cells; their antitumor reactivity may be enhanced by BCG or zoledronate in experimental settings (15). Among less abundant CD4^+^ T-cell subsets, Th17 cells have been detected at increased frequencies in bladder tumor tissue, supporting the presence of a type-17 inflammatory component within the local TIME (16). T follicular helper (Tfh) cells have also been characterized in bladder cancer-draining lymph nodes, suggesting a potential role in B-cell-associated immune organization, although their intratumoral distribution and function remain less well defined (17). NKT-cell populations have additionally been identified by single-cell profiling within urothelial bladder carcinoma, including exhaustion-associated transcriptional states (18). Collectively, these findings indicate that the bladder cancer immune landscape extends beyond the major T-cell, NK-cell, B-cell, and myeloid compartments, although the spatial distribution and functional significance of these less abundant populations remain less well characterized.

B cells and tertiary lymphoid structures (TLS) are similarly context dependent. Atypical B cells have been associated with tumor progression and poor BCG response in NMIBC (19). Recent studies have further characterized regulatory B cells (Bregs) as a heterogeneous immunosuppressive B-cell population in bladder cancer (20, 21). Raja et al. identified multiple Breg phenotypes, including IL-10^+^, TGF-β^+^, and IL-10^+^TGF-β^+^ B cells, with memory B cells showing the highest frequency of Breg phenotypes among the evaluated B-cell subsets (20). Zhou et al. further demonstrated increased CD19^+^IL-10^+^ Breg infiltration in bladder cancer tissues and showed that Bregs enhanced bladder cancer cell migration and invasion (21). In contrast, other B-cell populations can participate in antigen presentation and TLS-associated immune responses (22). Single-cell analysis of MIBC has further identified heterogeneous B-cell populations, including naïve, proliferating, interferon-stimulated, germinal-center-associated B cells and plasma cells, together with organized TLS containing B-cell follicles surrounded by T cells (22). These findings indicate that the biological relevance of B-cell infiltration depends on cellular phenotype and differentiation state, anatomical localization, TLS organization, and interactions with surrounding immune populations rather than on B-cell density alone. Where TLS maturation has been specifically assessed, early and secondary follicle-like TLS have shown different distributions across tissue compartments (23), further supporting the importance of TLS architecture and maturation. Accordingly, B-cell aggregates should not be classified simply as uniformly beneficial or suppressive; their clinical significance appears to depend on their cellular composition, organization, maturation, and local immune context.

The myeloid compartment provides a clear example of histologically compartmentalized macrophage phenotypes. TAMs are often described using an M1-like/M2-like framework, although this terminology represents a simplified phenotypic continuum rather than discrete functional states. TAMs can occupy heterogeneous and plastic transcriptional and functional states, and these states may vary according to local microenvironmental and therapeutic contexts (1, 4). Accordingly, M1-like and M2-like terminology is used here as descriptive shorthand rather than as a designation of fixed or mutually exclusive macrophage states. M1-like phenotypes have been associated with inflammatory antitumor programs, whereas M2-like phenotypes have been associated with immune suppression, angiogenesis, matrix remodeling, and invasion (1, 24). CD163 and CD204 are frequently used as markers of macrophage populations with M2-associated phenotypes in bladder cancer studies (24–26); however, expression of these markers alone does not demonstrate immunosuppressive biological activity. Consistent with this limitation, more recent phenotypic and single-cell studies have expanded the characterization of bladder cancer TAMs beyond conventional CD163- and CD204-based phenotyping (27, 28). Singh et al. reported increased TAM infiltration in urothelial bladder carcinoma and found that TAMs displayed both M1- and M2-associated phenotypic features, with the M2 phenotype positively associated with disease severity (28). Single-cell transcriptomic profiling further identified heterogeneous TAM populations, including SPP1^+^, FOLR2^+^, MKI67^+^, C1QC^+^, and LY6D^+^ macrophages, with SPP1^+^, FOLR2^+^, and C1QC^+^ populations showing relatively high M2-signature scores (27). Notably, SPP1^+^ macrophages showed functional remodeling from a phagocytic phenotype in NMIBC toward an angiogenesis-associated phenotype in MIBC, while FOLR2^+^ and SPP1^+^ macrophages were implicated in interactions contributing to a pro-angiogenic tumor microenvironment (27). Together, these findings further illustrate that bladder cancer TAMs comprise heterogeneous and plastic states that extend beyond a binary M1/M2 framework. In histological compartment-based analyses, studies have reported that macrophages are enriched in the tumor stroma and invasive fronts rather than being uniformly distributed within tumor nests (25, 26). This stromal preference is clinically important because it may support immune exclusion and tumor invasion even when lymphocytes are detectable elsewhere in the specimen.

MDSCs add another suppressive myeloid layer by inhibiting CD8^+^ T-cell, CD4^+^ T-cell, and NK-cell function through multiple immunosuppressive mechanisms (4, 5). PMN-MDSCs are enriched in bladder cancer tissues and can impair CD8^+^ T-cell function (29). However, the identification of MDSCs in human tumor tissue remains methodologically challenging. PMN-MDSCs and monocytic MDSCs (M-MDSCs) have been defined using different phenotypic marker combinations across studies, with substantial phenotypic overlap with conventional neutrophils and monocytes; therefore, cross-study comparisons should be interpreted according to the specific phenotypic and, where available, functional criteria used to define each MDSC subset (30).

DCs represent an antigen-presenting-cell component of the bladder cancer TIME and show non-uniform localization across histological compartments. Ayari et al. reported that mature CD83^+^ DCs were present in the papillary axis and underlying stroma, were rarely detected within tumor areas, and were most frequently observed within lymphoid aggregates (31).

A critical synthesis of these data leads to three practical conclusions. First, bladder cancer TIME is frequently compartmentalized: cytotoxic cells, regulatory lymphocytes, suppressive myeloid cells, and antigen-presenting structures do not occupy equivalent biological niches. Second, apparent contradictions between studies may arise from different sampling sites, disease stages, histological subtypes, antibodies, digital thresholds, or definitions of intratumoral versus stromal compartments. Third, compartment-resolved immune patterns are likely to be more clinically informative than bulk immune-cell counts because they can distinguish tumor-accessible immune infiltration with features associated with cytotoxic activity from infiltration that is spatially excluded or accompanied by exhaustion-associated, myeloid, or regulatory phenotypes.

### 2.2 Disease-state associations: stage, grade, and invasiveness

Disease-state associations are uneven across immune-cell populations. Among the more consistently reported associations in the reviewed literature are relationships between TAM enrichment, particularly M2-like or CD204^+^/CD163^+^ macrophage patterns, and higher stage, higher grade, or invasive behavior. Importantly, these observations derive mainly from mixed-stage bladder cancer cohorts rather than from a single disease category. Hanada et al. reported higher TAM counts in a cohort including both “superficial” and invasive bladder cancers, as classified in the original publication (32). Takeuchi et al. similarly found that CD163^+^/CD68^+^ M2-like TAMs were associated with pathological stage and histological grade in a mixed NMIBC/MIBC cohort (24), while Wang et al. reported that high stromal CD204^+^ TAM density correlated with advanced stage, high grade, and lymph-node metastasis in a mixed-stage urothelial bladder cancer cohort (25). More recent studies have extended these earlier observations using phenotypic and single-cell approaches (27, 28). Singh et al. reported that an M2-associated TAM phenotype characterized using CD206 and CD163 was positively associated with disease severity and clinicopathological parameters in urothelial bladder carcinoma (28). At single-cell resolution, Li et al. identified progression-associated remodeling of heterogeneous macrophage populations across adjacent tissue, NMIBC, and MIBC, including SPP1^+^ and FOLR2^+^ TAM states (27). Notably, SPP1^+^ TAMs shifted from a predominantly phagocytic phenotype in NMIBC toward an angiogenesis-associated phenotype in MIBC, providing evidence of macrophage-state remodeling accompanying progression to muscle-invasive disease (27). These convergent findings support the interpretation that macrophage alterations associated with more advanced bladder cancer involve not only enrichment of conventionally defined M2-like TAMs but also broader phenotypic and transcriptional remodeling.

For other immune populations, disease-state associations are more heterogeneous and context dependent. Recent studies have identified regulatory Bregs as one such disease-associated population. Raja et al. found that high-grade bladder tumors showed increased infiltration of Bregs, including IL-10^+^, TGF-β^+^, and IL-10^+^TGF-β^+^ populations, with memory B cells showing particularly prominent Breg phenotypes (20). The same study further reported associations between TLS frequency, disease severity, and IL-10^+^ Breg-cell counts (20). Zhou et al. likewise reported increased CD19^+^IL-10^+^ Breg infiltration in bladder cancer and associated Breg enrichment with more advanced disease (21). CD8^+^ T-cell findings also vary by disease setting. In muscle-invasive urothelial carcinoma, Sharma et al. observed similar intratumoral CD8^+^ detection rates across earlier and more advanced disease (7), whereas Rejeb et al., in a small invasive urothelial carcinoma cohort spanning ≥pT1 disease, found no significant correlation between CD8^+^ density and tumor stage (8). Treg data are likewise context dependent: Miyake et al. associated high Treg counts with T1 and high-grade NMIBC (11), whereas Horn et al., in an invasive bladder cancer cohort, did not identify a clear association with stage or grade (33). FATP2-high/RIPK3-low PMN-MDSC-associated patterns were reported in more advanced bladder cancer, with higher FATP2 and lower RIPK3 expression in stage III/IV than in stage I/II disease (29), further suggesting that suppressive myeloid programs may vary with tumor stage.

A critical interpretation is therefore that differences in stage and grade are associated not simply with differences in immune-cell abundance, but also with differences in immune architecture. Across the reviewed cohorts, more invasive disease was more often associated with stromal myeloid enrichment and regulatory-cell accumulation. These cross-sectional associations do not establish that such immune changes causally drive tumor progression, but they suggest that CD8^+^ T-cell density alone may incompletely capture stage-associated differences in immune architecture. Composite patterns incorporating CD8^+^ T-cell phenotype, Treg burden, TAM phenotype, and stromal localization may therefore be more biologically informative. However, these conclusions remain limited by differences in antibody markers, scoring methods, tumor compartments analyzed, and the mixture of NMIBC and MIBC cohorts across studies.

### 2.3 Treatment-associated remodeling of the bladder cancer TIME

This section summarizes treatment-associated remodeling of the bladder cancer TIME. The purpose is not to claim that post-treatment immune changes are already validated predictors of prognosis, but to describe how therapeutic pressure can modify immune architecture and functional states. The available evidence includes paired pre-/post-treatment human analyses, unpaired treatment-exposed versus untreated comparisons, sequential tissue studies, preliminary clinical reports, and preclinical models. These designs do not provide equivalent inferential strength. Paired longitudinal human specimens provide the most direct evidence of treatment-associated remodeling, particularly when immune changes are linked to clinical outcomes. Unpaired treated-versus-untreated comparisons provide supportive but less direct evidence because between-patient differences cannot be excluded, whereas preclinical models primarily provide mechanistic evidence and conference-only data should be considered preliminary and hypothesis-generating. Importantly, treatment-associated immune changes are not uniformly beneficial, because the same treatment may increase effector-cell infiltration while simultaneously inducing exhaustion, checkpoint upregulation, or suppressive myeloid expansion.

BCG provides one of the clearer clinical examples of treatment-associated immune changes in bladder cancer, although the available studies differ in BCG exposure and sampling context. Lim et al. evaluated immune changes during and after BCG induction and reported that responders showed increased CD4^+^FOXP3^−^ T cells and CD8^+^PD-1^−^ T cells, whereas non-responders showed an increase in CD8^+^PD-1^+^ T cells characterized as exhausted in the original study, together with increased CD8^+^ cell numbers without corresponding CD69 activation (34). Responders were defined by the absence of recurrence at 24 months after the last BCG exposure, and maintenance treatment varied among patients. Hashizume et al. examined paired pre- and post-treatment specimens specifically from BCG-resistant NMIBC and reported increased PD-L1 expression after BCG (35). By contrast, Maas et al. analyzed sequential tumors from both BCG-treated and BCG-naïve high-risk NMIBC patients and found that BCG exposure was associated with changes in PD-L1 expression (36). These differences in treatment exposure, prior BCG status, response definitions, and sampling context should be considered when comparing post-BCG immune remodeling across studies. Collectively, these studies indicate that BCG-associated immune remodeling is heterogeneous and that treatment outcome cannot be inferred from an increase in immune-cell abundance alone. Rather, the clinical relevance of post-BCG immune changes appears to depend on the functional state of infiltrating lymphocytes and the concurrent development of checkpoint-associated immune adaptation (34–36). These observations suggest that the balance between treatment-associated effector activation and compensatory immune suppression may contribute to differences in BCG response and resistance.

Paired human studies provide direct evidence of NAC-associated immune remodeling, although such longitudinal evidence remains less extensive than that available for BCG. Wu et al. examined 54 paired MIBC specimens before and after NAC and found significant decreases in CD68^+^ and CD68^+^PD-L1^−^ TAM infiltration after treatment, whereas CD68^+^PD-L1^+^ TAMs and the evaluated T-cell populations showed no significant paired changes (37). In a broader platinum-based chemotherapy setting, Chelushkin et al. analyzed paired pre- and post-treatment samples from 95 patients with urothelial bladder cancer and reported increased intratumoral CD8^+^ T-cell proportions and PD-L1^+^ immune cells after chemotherapy, together with increased fibroblast TGF-β signaling and greater spatial separation between immune cells and cancer cells (38). These findings indicate that platinum-based chemotherapy can remodel both immune-cell composition and stromal-spatial organization, with immune-activating and potentially immunosuppressive changes occurring in parallel. Beyond the clinical cisplatin-based NAC setting, experimental intravesical chemotherapy models also suggest that chemotherapy can modulate CD8^+^ T-cell activation and regulatory immune features (39). However, this experimental intravesical setting is biologically and therapeutically distinct from systemic cisplatin-based NAC. Evidence from adjuvant or metastatic platinum-treated populations should likewise be interpreted separately rather than extrapolated from NAC studies.

ICI-associated remodeling is also context dependent, but the available bladder cancer evidence is not uniformly based on matched pre-/post-treatment specimens. In urothelial cancer, van Dijk et al. compared untreated tumors with tumors exposed to preoperative anti-PD-1/CTLA-4 therapy and reported increased CD8^+^PD-1^+^ T cells in ICI-treated tumors, as well as treatment-associated differences in TLS architecture (23). A preliminary conference report from the neoadjuvant pembrolizumab PURE-01 setting directly compared pre- and post-immunotherapy specimens and suggested that non-responders may still show increased CD8 ^+^ and CD20 ^+^ cell densities after treatment. Nevertheless, it suggests that post-treatment increases in immune-cell density may reflect treatment-associated immune remodeling and should not, by themselves, be interpreted as evidence of an effective antitumor response. preliminary and hypothesis-generating (40). These remodeling observations derive primarily from preoperative or neoadjuvant ICI settings and should not be generalized to adjuvant or metastatic ICI treatment, which represent distinct therapeutic contexts.

Radiotherapy-associated remodeling remains less well characterized because paired human tumor samples before and after radiotherapy are limited. Preclinical bladder cancer models of radiation with or without checkpoint inhibition suggest that irradiation can enhance CD8^+^ T-cell activity while also inducing compensatory immunosuppressive changes, including PD-L1 upregulation and expansion of MDSCs, TAMs, or Tregs (41). Separately, Wu et al. evaluated a retrospective cohort of MIBC patients treated with definitive concurrent chemoradiotherapy rather than radiotherapy alone, together with experimental irradiation models in which irradiation upregulated tumor-cell PD-L1 expression, supporting radiation-associated checkpoint modulation (42). These findings should therefore not be interpreted as evidence that radiotherapy alone, concurrent chemoradiotherapy, and other bladder-preserving regimens produce equivalent immune effects.

Overall, treatment should be viewed as an ecological perturbation of the bladder cancer TIME, but the strength of evidence differs across therapeutic modalities. BCG currently has relatively stronger human evidence for treatment-associated immune remodeling, while paired human studies also provide direct evidence of immune remodeling after NAC or broader platinum-based chemotherapy. Evidence for ICI therapy and radiotherapy remains more heterogeneous and includes treatment-exposed versus untreated comparisons, preliminary pre-/post-treatment analyses, and preclinical models. Collectively, these studies indicate that treatment-associated TIME remodeling is context dependent and may involve concurrent changes in immune-cell composition, functional state, checkpoint expression, and stromal or spatial organization. Further longitudinal studies using matched human specimens are needed to define the consistency, temporal dynamics, and clinical relevance of these treatment-associated changes. The principal study-level evidence for treatment-associated TIME remodeling across therapeutic modalities is summarized in **Table 1**.

**Table 1:** Study-level evidence for treatment-associated remodeling of the bladder cancer tumor immune microenvironment.

Study (ref.)	Clinical setting/study design	Main finding	Supported conclusion/inferential strength
Lim et al. (34)	BCG-treated bladder cancer; human clinical immune profiling	Responders and non-responders showed distinct T-cell phenotypic patterns, including activation- and exhaustion-associated states after BCG exposure.	Human clinical evidence supporting BCG-associated immune-state differences. Associations with response do not by themselves establish treatment-specific predictive value.
Hashizume et al. (35)	BCG-treated NMIBC; human tumor study	BCG exposure was associated with PD-L1 upregulation and immune changes observed in non-responders.	Supports BCG-associated checkpoint modulation in human tumors, but does not by itself show that PD-L1 change predicts treatment benefit.
Maas et al. (36)	High-risk NMIBC receiving BCG; sequential human tumor tissue analysis	BCG exposure was associated with changes in PD-L1 expression in tumor and inflammatory cells across sequential tumor tissues.	Relatively direct human evidence of BCG-associated TIME remodeling because sequential human specimens were evaluated.
Wu et al. (37)	MIBC treated with NAC; paired pre- and post-treatment human specimens	CD68^+^ and CD68^+^PD-L1^−^ TAM infiltration decreased significantly after NAC, whereas CD68^+^PD-L1^+^ TAMs and the evaluated T-cell populations showed no significant paired changes.	Direct longitudinal human evidence of NAC-associated macrophage remodeling. The paired design supports treatment-associated change but does not by itself establish treatment-specific predictive utility.
Chelushkin et al. (38)	Urothelial bladder cancer treated with platinum-based chemotherapy; paired pre- and post-treatment human samples	Chemotherapy was associated with increased intratumoral CD8^+^ T-cell proportions and PD-L1^+^ immune cells, increased fibroblast TGF-β signaling, and greater spatial separation between immune and tumor cells.	Direct longitudinal human evidence that platinum-based chemotherapy is associated with concurrent immune and stromal-spatial remodeling. This broader platinum-treated setting should not be interpreted as equivalent to cisplatin-based NAC.
Chen et al. (39)	Experimental intravesical chemotherapy; preclinical bladder cancer study	Intravesical chemotherapy modulated CD8^+^ T-cell activation and regulatory immune features.	Mechanistic evidence of intravesical chemotherapy-associated immune modulation; this experimental setting is distinct from systemic cisplatin-based NAC, adjuvant chemotherapy, or metastatic platinum treatment.
van Dijk et al. (23)	Preoperative anti-PD-1/CTLA-4 exposure; unpaired treated-versus-untreated human tumor comparison	ICI-exposed tumors showed increased CD8+PD-1+ T cells and treatment-associated differences in TLS architecture.	Supports treatment-associated immune differences, but causal treatment-induced remodeling is less certain than in paired longitudinal studies because between-patient differences cannot be excluded.
Cattaneo et al. (40)	Neoadjuvant pembrolizumab; paired pre-/post-treatment human specimens; preliminary conference report	CD8+ and CD20+ cell densities could increase after treatment even in non-responders.	Direct longitudinal evidence of ICI-associated immune change, but the evidence remains preliminary and hypothesis-generating because it is conference-only.
Rompre-Brodeur et al. (41)	Radiation +/- checkpoint inhibition; primarily preclinical bladder cancer models	Radiation enhanced antitumor immune activity while also inducing compensatory suppressive immune changes.	Mechanistic evidence for radiation-induced immune modulation; insufficient by itself to establish longitudinal TIME remodeling in human tumors.
Wu et al. (42)	Experimental irradiation models together with a retrospective MIBC cohort treated with definitive concurrent chemoradiotherapy	Irradiation upregulated tumor-cell PD-L1 expression in experimental models.	Supports mechanistic evidence of radiation-associated checkpoint modulation; the accompanying clinical evidence derives from concurrent chemoradiotherapy rather than radiotherapy alone and does not establish paired human TIME remodeling.

Inferential strength is considered relative to the question of treatment-associated TIME remodeling. Paired or longitudinal human specimens provide the most direct evidence of treatment-associated change, whereas unpaired clinical comparisons provide supportive but less direct evidence. Preclinical studies primarily establish mechanistic plausibility, and conference-only evidence should be considered preliminary and hypothesis-generating.

### 2.4 Baseline TIME characteristics associated with treatment response and prognosis

The biomarker evidence reviewed in this section focuses primarily on untreated or pre-treatment tumor samples. Where studies used peri-treatment or post-treatment samples, this is stated explicitly or interpreted with caution. Most available studies are treatment-specific clinical cohorts, retrospective biomarker analyses, or molecular profiling studies. Such designs can establish associations between baseline immune features and treatment response or clinical outcomes, but they do not by themselves demonstrate treatment-induced TIME remodeling or treatment-specific predictive value. As defined above, predictive utility requires comparative evidence demonstrating biomarker-by-treatment interaction. Transcriptomic, single-cell, and other high-dimensional approaches can provide detailed biological characterization, but their clinical significance depends on the underlying cohort design and independent outcome validation. The purpose of this section is not to identify a single universal biomarker, but to evaluate how baseline immune-cell abundance, histological compartment-based localization, and functional state are associated with treatment response and prognosis in specific therapeutic settings. The study-level evidence on baseline TIME biomarkers, treatment response, prognosis, and high-dimensional immune characterization is summarized in **Table 2**.

**Table 2:** Study-level evidence informing baseline TIME biomarkers, treatment response, prognosis, and high-dimensional immune characterization in bladder cancer.

Study (ref.)	Clinical setting/study design	Main finding	Supported conclusion/inferential strength
Kates et al. (2)	BCG-treated NMIBC; clinical biomarker study	PD-1/PD-L1-related adaptive immune-resistance patterns were associated with BCG-related outcomes.	Supports treatment-response-associated immune patterns within a BCG-treated cohort; treatment-specific predictive value is not established by association alone.
Pichler et al. (10)	BCG-treated bladder cancer; clinical immune profiling	Th1-associated inflammatory activity and lower suppressive signaling were associated with clinical response to BCG.	Supports a treatment-response-associated immune phenotype in BCG-treated patients.
Miyake et al. (11)	BCG-treated NMIBC; long-term follow-up clinical cohort	Treg- and TAM-related suppressive features were associated with poor BCG response and shorter recurrence-free survival.	Provides treatment-response-associated and prognostic evidence within a treated cohort; it does not demonstrate treatment-effect modification.
Yolmo et al. (19)	NMIBC; translational B-cell/TLS study	Atypical B-cell and B-cell-dominant immune programs were associated with tumor progression and poor BCG response.	Supports treatment-response-associated biological stratification; causal and treatment-specific predictive interpretations require further validation.
Lima et al. (26)	BCG-treated bladder cancer; clinical biomarker study	Stromal M2-like TAM predominance was associated with BCG failure.	Supports an adverse treatment-response-associated macrophage pattern in BCG-treated tumors.
Ayari et al. (31)	BCG-treated bladder cancer; pretreatment clinical biomarker study	High pretreatment CD68+ TAM and CD83+ tumor-infiltrating dendritic-cell infiltration was associated with poorer BCG response.	Supports a baseline treatment-response association, but not strict treatment-specific predictive utility.
Drachneris et al. (43)	BCG-treated NMIBC; histological tumor–stroma interface biomarker study	The CD8+ cell-density gradient across the tumor-stromal interface was associated with recurrence-free survival after BCG.	Supports a compartment-based prognostic and treatment-response-associated relationship in BCG-treated patients.
Suriano et al. (44)	BCG-treated NMIBC; clinical macrophage biomarker study	TAM polarization was associated with BCG efficacy and recurrence risk.	Supports clinical relevance of macrophage phenotype in the BCG setting, but does not demonstrate direct BCG-induced TAM reprogramming or strict predictive value.
Fukiage et al. (45)	BCG-treated bladder carcinoma *in situ*; clinical biomarker study	Higher peritumoral Treg density was associated with BCG non-response.	Supports an association between peritumoral Treg enrichment and BCG non-response.
Andreasen et al. (46)	BCG-treated NMIBC; PD-1/PD-L1 biomarker study	Pretreatment PD-1/PD-L1-related expression patterns were associated with BCG-related outcomes.	Supports response-associated checkpoint patterns; treatment-specific predictive utility is not established.
de Jong et al. (47)	High-risk NMIBC; clinical PD-L1 biomarker cohort	Baseline PD-L1 alone showed limited ability to distinguish BCG responders from non-responders.	Provides evidence that baseline PD-L1 alone has limited value for BCG response stratification in this setting.
Mendoza-Valderrey et al. (3)	MIBC treated with cisplatin-based NAC; clinical immune biomarker study	B-cell and CD3+ T-cell enrichment was associated with improved response, whereas inhibitory immune features were associated with non-response.	Supports treatment-response-associated immune stratification within an NAC-treated cohort; it does not establish strict predictive value.
Baras et al. (48)	MIBC treated with cisplatin-based NAC; pretreatment TIL biomarker study	A higher pretreatment CD8/Treg ratio was associated with response, whereas higher Treg proportions were associated with resistance.	Supports a baseline treatment-response-associated biomarker within an NAC-treated cohort.
Nassif et al. (49)	Localized urothelial carcinoma treated with NAC; Immunoscore study	Higher CD3+ and CD8+ Immunoscore-based infiltration was associated with longer time to recurrence, overall survival, and 5-year recurrence-free survival.	Primarily supports prognostic and outcome-associated stratification in NAC-treated patients.
Ikarashi et al. (50)	MIBC in the NAC setting; pretreatment TIME study	Ki-67-negative CD8+ T cells with high CD204+ TAM infiltration were associated with resistance, whereas proliferative CD8+ cells with lower TAM burden were associated with more favorable outcomes.	Supports baseline immune-response stratification and prognostic association; it does not establish treatment-effect modification.
Wu et al. (37)	MIBC treated with NAC; clinical macrophage study	Stromal CD68+ TAM-related patterns were associated with poorer disease-free survival.	Supports prognostic association in an NAC-treated population; dynamic immune changes should not automatically be interpreted as predictive evidence.
Yan et al. (51)	MIBC; NKG2A/PD-L1 panel evaluated in chemotherapy and PD-L1 blockade settings	NKG2A/PD-L1 co-expression was associated with clinical benefit across treatment contexts.	Supports multi-marker treatment-response stratification; strict treatment-specific predictive interpretation requires appropriate comparative treatment evidence.
Su et al. (52)	MIBC; CDK6/PD-L1/TMB integrative molecular score evaluated across platinum-based chemotherapy and PD-1/PD-L1 blockade cohorts	The composite score was associated with response to platinum-based chemotherapy and PD-1/PD-L1 blockade.	Supports molecular response stratification across treatment settings; independent validation and formal treatment-interaction testing are required for strict predictive interpretation.
Eckstein et al. (53)	MIBC after radical cystectomy and adjuvant chemotherapy; gene-expression study	A cytotoxic T-cell-related gene-expression signature was associated with improved survival.	Provides transcriptomic prognostic association in a treated population; it does not by itself establish treatment-specific predictive utility.
Balar et al. (54)	Cisplatin-ineligible locally advanced/unresectable or metastatic urothelial carcinoma; KEYNOTE-052, multicenter single-arm phase II study	Higher baseline PD-L1 positivity was associated with pembrolizumab efficacy.	Treatment-response-associated evidence from a single-arm ICI cohort; prognostic and predictive effects cannot be separated without a comparator.
Necchi et al. (55)	MIBC receiving neoadjuvant pembrolizumab before radical cystectomy; PURE-01, single-arm phase II study	Baseline PD-L1 positivity was associated with pathological response.	Supports response-associated stratification within pembrolizumab-treated patients, but not strict treatment-specific predictive value.
Rosenberg et al. (56)	Locally advanced or metastatic urothelial carcinoma after platinum-based chemotherapy; atezolizumab, multicenter single-arm phase II study	Higher PD-L1 expression on tumor-infiltrating immune cells was associated with response and survival.	Supports treatment-response-associated and prognostic associations within an ICI-treated cohort; comparative evidence is lacking for strict predictive value.
Li et al. (57)	Bladder cancer; molecular/TME subtype analysis	Immune-inflamed TME subtypes were associated with prognosis and anti-PD-L1 response.	Provides molecular/TME-based response and prognostic stratification; clinical predictive interpretation requires appropriately designed comparative validation.
Liang et al. (58)	Bladder cancer; immunophenotype profiling	Distinct tumor immunophenotypes were associated with immunotherapy response.	Supports immune-response stratification, but association alone does not establish treatment-specific predictive utility.
Zeng et al. (59)	Urothelial cancer; macrophage/TME profiling	M1-TAM-related inflammatory states discriminated anti-PD-L1 responders from non-responders.	Supports treatment-response-associated immune stratification; comparative treatment evidence is required for strict predictive classification.
Wu et al. (42)	MIBC treated with definitive concurrent chemoradiotherapy; retrospective clinical biomarker analysis	Tumor PD-L1 expression was associated with radiation response and clinical outcome.	Supports treatment-response-associated and prognostic evidence in the concurrent chemoradiotherapy setting; these findings should not be generalized to radiotherapy alone
Marcq et al. (60)	MIBC treated with radiation-based bladder-preserving therapy; clinical biomarker study	Higher pretreatment PD-L1 expression in immune cells was associated with a higher complete-response rate and better overall survival.	Supports baseline treatment-response-associated and prognostic evidence in a radiation-treated cohort.
Smith et al. (61)	MIBC; BCON phase III randomized trial comparing radiotherapy alone with radiotherapy plus hypoxia-modifying carbogen and nicotinamide; baseline biomarker analysis	Low CD8+ T-cell counts were associated with benefit from hypoxia-modifying therapy.	Supports treatment-benefit stratification within the randomized hypoxia-modification setting; the finding should not be generalized to radiotherapy or chemoradiotherapy settings more broadly.
Liu et al. (6)	MIBC; single-cell RNA-sequencing study	Single-cell analysis characterized immune-cell heterogeneity and transcriptional states related to T-cell infiltration.	Provides high-resolution biological and mechanistic characterization; treatment-response or prognostic relevance requires outcome-linked clinical validation.
Yuan et al. (22)	MIBC; single-cell sequencing study of B-cell populations and tertiary lymphoid structures	Single-cell analysis identified heterogeneous B-cell populations and organized TLS containing B-cell follicles surrounded by T cells.	Provides high-dimensional biological characterization of B-cell and TLS heterogeneity; treatment-response or prognostic implications require outcome-linked clinical validation.

Across BCG-treated NMIBC cohorts, unfavorable associations have more often involved regulatory, checkpoint-associated, or exhaustion-associated marker patterns in particular histological compartments rather than simply low immune infiltration. The CD8^+^ density gradient across the tumor-stromal interface has been associated with recurrence-free survival after BCG immunotherapy (43). High pre-treatment CD68^+^ TAM and CD83^+^ tumor-infiltrating dendritic-cell have been associated with poorer BCG response (31), and stromal CD163^+^ macrophage predominance, interpreted in the original study as an M2-like phenotype, has been linked to BCG failure and shorter RFS (26). Suriano et al. reported that TAM polarization was associated with the efficacy of BCG instillation and recurrence risk, but this study should be interpreted primarily as evidence that macrophage phenotype is clinically relevant within the BCG-treated setting, rather than as proof that BCG directly reprograms TAMs in matched human tumors or that macrophage phenotype is a treatment-specific predictive biomarker (44). Tregs and CD204^+^ TAMs were associated with poor response and shorter RFS in a Japanese BCG-treated NMIBC cohort (11). In a BCG-treated carcinoma *in situ* cohort, higher peritumoral Treg density within a 20-μm region around the lesion was associated with non-response (45). At the same time, Th1-associated inflammatory immune activity and lower IL-10-related suppressive signaling were associated with response (10), whereas B-cell-dominant TLS adjacent to epithelium and exhaustion-related signals were more common in non-responders (19). PD-L1/CD8 colocalization and low pre-treatment PD-1/PD-L1 expression patterns have been associated with BCG response-related outcomes in selected studies (2, 46), whereas baseline PD-L1 alone showed limited ability to distinguish BCG responders from non-responders in a larger treated cohort (47). Taken together, across the reviewed BCG-treated cohorts, differences in treatment outcome, including response, resistance, and non-response, appear to be associated with the overall balance, functional state, and spatial organization of effector and suppressive immune features within the baseline or pretreatment TIME, rather than with any single immune-cell population. Functionally, an effector-oriented and tumor-accessible immune context may be more compatible with effective BCG-induced antitumor activity, whereas regulatory- or checkpoint-dominant immune states have more often been associated with resistance or non-response. Thus, currently reported BCG-associated immune features are best interpreted as treatment-response-associated or prognostic patterns rather than established treatment-specific predictive biomarkers. Their interpretation should also account for heterogeneity in BCG treatment exposure and adequacy, prior treatment status, response or failure definitions, recurrence versus progression endpoints, and disease composition, including carcinoma *in situ*.

In localized MIBC treated with cisplatin-based NAC, a recurring biomarker theme is the balance between cytotoxic immune competence and suppressive myeloid or regulatory features. In this neoadjuvant setting, a higher pretreatment CD8/Treg ratio was associated with response, whereas higher Treg proportions were associated with resistance (48). Higher Immunoscore-based CD3^+^ and CD8^+^ infiltration was associated with longer time to recurrence, overall survival, and 5-year RFS in localized urothelial carcinoma treated with NAC (49). However, high CD8^+^ density alone is not sufficient. In pretreatment MIBC specimens from the NAC setting, Ikarashi et al. showed that non-proliferative Ki-67− CD8^+^ T cells combined with CD204^+^ TAM accumulation indicated a suppressive immune state associated with chemotherapy resistance, whereas proliferative CD8^+^ T-cell accumulation with low CD204^+^ TAM infiltration was more favorable (50). CD68^+^ stromal TAMs were associated with poorer DFS (37), while B-cell and CD3^+^ T-cell enrichment correlated with improved response and inhibitory protein enrichment correlated with non-response (3). Evidence from non-neoadjuvant platinum-based settings should be considered separately. Yan et al. reported that NKG2A/PD-L1 co-expression was associated with clinical benefit across distinct cohorts receiving cisplatin-based adjuvant chemotherapy or PD-L1 blockade (51). Su et al. found that a composite CDK6/PD-L1/TMB score was associated with response to platinum-based chemotherapy as well as PD-1/PD-L1 blockade (52). In a postoperative adjuvant setting, Eckstein et al. reported that a cytotoxic T-cell-related gene-expression signature was associated with improved survival in MIBC patients after radical cystectomy and adjuvant chemotherapy (53). These findings should be interpreted within their respective therapeutic contexts rather than pooled with biomarker evidence derived from cisplatin-based NAC cohorts. Although these studies support the potential value of multi-marker immune stratification, most evaluate associations within treated cohorts and therefore do not, by themselves, establish treatment-specific predictive value.

For ICI therapy, baseline PD-L1 associations have been reported across distinct disease settings and should therefore be interpreted separately. In locally advanced, unresectable, or metastatic urothelial carcinoma, KEYNOTE-052 associated higher baseline PD-L1 positivity with pembrolizumab efficacy (54), while Rosenberg et al. reported associations between higher PD-L1 expression on tumor-infiltrating immune cells and response or survival in locally advanced or metastatic urothelial carcinoma after platinum-based chemotherapy (56). By contrast, PURE-01 evaluated neoadjuvant pembrolizumab in muscle-invasive bladder cancer before radical cystectomy and reported an association between baseline PD-L1 positivity and pathological response (55). More recent randomized evidence from IMvigor130 showed that atezolizumab plus platinum-based chemotherapy did not achieve a significant overall-survival benefit over chemotherapy alone; because of the hierarchical study design, the atezolizumab monotherapy comparison was descriptive and exploratory and likewise did not show an overall-survival improvement in the intention-to-treat population (62). These findings do not necessarily contradict the earlier associations between baseline PD-L1 expression and response or survival; rather, they indicate that an association between baseline PD-L1 expression and outcome within atezolizumab-treated patients does not by itself establish PD-L1 as a treatment-specific predictor of survival benefit over chemotherapy. Accordingly, the earlier single-arm PD-L1 associations should primarily be interpreted as treatment-response-associated findings rather than as definitive evidence of treatment-specific predictive value. More broadly, ICI response appears to be associated with an inflamed and treatment-permissive immune state rather than with PD-L1 positivity alone. TME subtypes enriched for CD8^+^ T cells, activated CD4^+^ memory T cells, NK cells, and M1 macrophages appear more responsive to anti-PD-L1 therapy (57, 58). Zeng et al. suggested that M1-TAM infiltration may better discriminate responders from non-responders within an anti-PD-L1-treated cohort than PD-L1 immunohistochemistry, CD8 markers, or T-cell inflammatory gene-expression profiles (59). Yan et al. further showed that the NKG2A/PD-L1 co-expression panel was associated with clinical benefit from PD-L1 blockade in a metastatic urothelial carcinoma cohort (51). Collectively, across these distinct therapeutic settings, the available studies support the use of baseline immune architecture for treatment-response stratification, but comparative treatment studies with formal biomarker-by-treatment interaction testing are required before such features can be considered predictive biomarkers in the strict sense.

Radiotherapy-related biomarkers should also be interpreted within specific treatment contexts. In Wu et al., tumor-cell PD-L1 expression was associated with response and outcome in MIBC patients receiving definitive concurrent chemoradiotherapy (42). By contrast, Marcq et al. evaluated pretreatment PD-L1 expression in MIBC treated with radiation-based bladder-preserving therapy and found that higher PD-L1 expression in immune cells was associated with a higher complete-response rate and better overall survival (60). In the BCON trial, patients were randomized to radiotherapy alone or radiotherapy with hypoxia-modifying carbogen plus nicotinamide, and low CD8^+^ T-cell counts were associated with benefit from hypoxia modification (61). Therefore, the apparently divergent PD-L1 and CD8-related findings may reflect not only differences in the cellular compartment assessed, but also differences in treatment setting. Radiotherapy alone, concurrent chemoradiotherapy, hypoxia-modifying treatment, and multimodal bladder-preserving therapy should not be regarded as biologically interchangeable contexts.

Taken together, the evidence across BCG, platinum-based chemotherapy, immune checkpoint inhibition, and radiotherapy indicates that the clinical interpretation of baseline TIME features is strongly treatment-context dependent. Across these settings, single immune markers are generally less informative than composite assessment incorporating immune-cell phenotype, histological compartment-based localization, functional state, and therapeutic context. However, most reported TIME features currently represent treatment-response-associated or prognostic relationships rather than validated treatment-specific predictive biomarkers. Composite TIME-based models therefore remain investigational, and their incremental value beyond established clinicopathological and molecular biomarkers requires prospective validation. Treatment-associated immune changes described in Section 2.3 should additionally be evaluated longitudinally and should not be conflated with baseline or pretreatment biomarker associations.

### 2.5 Critical synthesis, limitations, and future directions

Four integrative principles emerge from the evidence reviewed in Sections 2.1–2.4. First, immune-cell abundance alone is insufficient to characterize the bladder cancer TIME. Its biological and clinical interpretation depends on histological compartment-based localization, cellular phenotype, and functional state. Cell identity, individual marker expression, inferred phenotype, and directly demonstrated biological activity should therefore be regarded as related but non-equivalent levels of evidence. Conventional compartment-based localization should also be distinguished from high-dimensional spatial profiling, which provides a different level of information on multicellular organization and cell–cell relationships.

Second, TIME characteristics vary with disease state and should be interpreted as differences in immune architecture rather than as simple changes in immune-cell abundance. Across the reviewed cohorts, associations with stage, grade, and invasiveness differ among immune-cell populations and are influenced by histological compartment, disease composition, and study design. Because much of this evidence is cross-sectional and derived from heterogeneous NMIBC and MIBC cohorts, these patterns should be interpreted as disease-state associations rather than as evidence of a causal or longitudinal evolution of the TIME.

Third, baseline or pretreatment immune architecture and treatment-associated remodeling represent distinct but complementary dimensions of the TIME. Baseline features may provide prognostic or treatment-response-associated information, whereas post-treatment changes describe dynamic immune adaptation. The strength of inference depends on study design: observational human cohorts primarily support associations, paired longitudinal studies provide stronger evidence of treatment-associated change, and experimental models provide mechanistic evidence. These evidence types should not be treated as equivalent. Likewise, treatment-response-associated findings within a treated cohort should not be considered treatment-specific predictive biomarkers unless comparative evidence demonstrates biomarker-by-treatment interaction. Interpretation should also account for disease setting, treatment modality, treatment exposure and adequacy, prior treatment status, sampling time point, and clinical endpoint.

Fourth, future biomarker development should move beyond single markers toward standardized, composite, and context-specific immune assessment. The current evidence base is limited by retrospective study designs, modest sample sizes, heterogeneous patient populations, variable marker definitions and cutoffs, inconsistent histological compartment annotation, and methodological differences among immunohistochemistry, bulk transcriptomics, single-cell approaches, and high-dimensional spatial profiling. Future studies should prioritize prospective and longitudinal designs with matched pre- and post-treatment specimens, standardized reporting of histological compartments, integration of complementary molecular and spatial approaches, and independent validation. Establishing treatment-specific predictive utility will additionally require comparative treatment cohorts with formal testing of biomarker-by-treatment interactions.

From a therapeutic perspective, the expanding checkpoint landscape also suggests opportunities beyond the PD-1/PD-L1 axis. Additional inhibitory checkpoints, including LAG-3, TIM-3, TIGIT, and B7-H4, have emerged as potential therapeutic targets in bladder cancer. In MIBC, high stromal LAG-3^+^-cell infiltration was associated with an immunoevasive microenvironment, dysfunctional CD8^+^ T cells, and increased expression of multiple inhibitory checkpoints, including PD-1, PD-L1, TIM-3, and TIGIT (63). TIM-3 and TIGIT have also been identified as candidate checkpoint targets on T and NK cells in bladder cancer, with functional evidence showing that TIM-3 blockade can enhance NK-cell effector activity (64). More recently, B7-H4 was found to be upregulated in urothelial carcinoma, and combined B7-H4/PD-L1 blockade enhanced IFN-γ and granzyme B production by CD8^+^ T cells ex vivo (65). Together, the coexistence of multiple inhibitory pathways and the functional effects observed with checkpoint co-blockade provide a biological rationale for combination strategies beyond PD-1/PD-L1 alone; however, their clinical benefit in bladder cancer remains to be established in prospective trials.

## 3 Conclusions

In summary, the bladder cancer TIME should be interpreted as a histologically compartmentalized and treatment-modifiable immune ecosystem rather than as a static collection of immune-cell markers. Current evidence indicates that several baseline or pretreatment TIME features show promising and biologically plausible associations with treatment response and prognosis, but such associations should not be equated with demonstrated clinical utility. Most TIME-based biomarkers discussed in this review remain investigational and have not yet demonstrated sufficient analytical validity, clinical validity, or clinical utility to support routine treatment selection. Their incremental value beyond established clinicopathological and molecular biomarkers also remains to be demonstrated in prospective studies. Treatment-associated remodeling provides mechanistic and descriptive insight, whereas its additional association with clinical outcomes requires validation in longitudinal cohorts with matched pre- and post-treatment specimens. Establishing treatment-specific predictive utility will additionally require comparative treatment designs with formal biomarker-by-treatment interaction testing.
